# Supplementary material for: The interactome of intact mitochondria by cross-linking mass spectrometry provides evidence for coexisting respiratory supercomplexes
Source: Mol Cell Proteomics. 2017 Dec 8;17(2):216–32. doi: 10.1074/mcp.RA117.000470 (PMC5795388; doi:10.1074/mcp.RA117.000470)
Supplement: Supplemental Data [file supp_RA117.000470_133922_0_supp_23978_fzffwf.zip › spectra_annotation/mito_spectra_annotation/406-1-3-1-6-1.pdf]

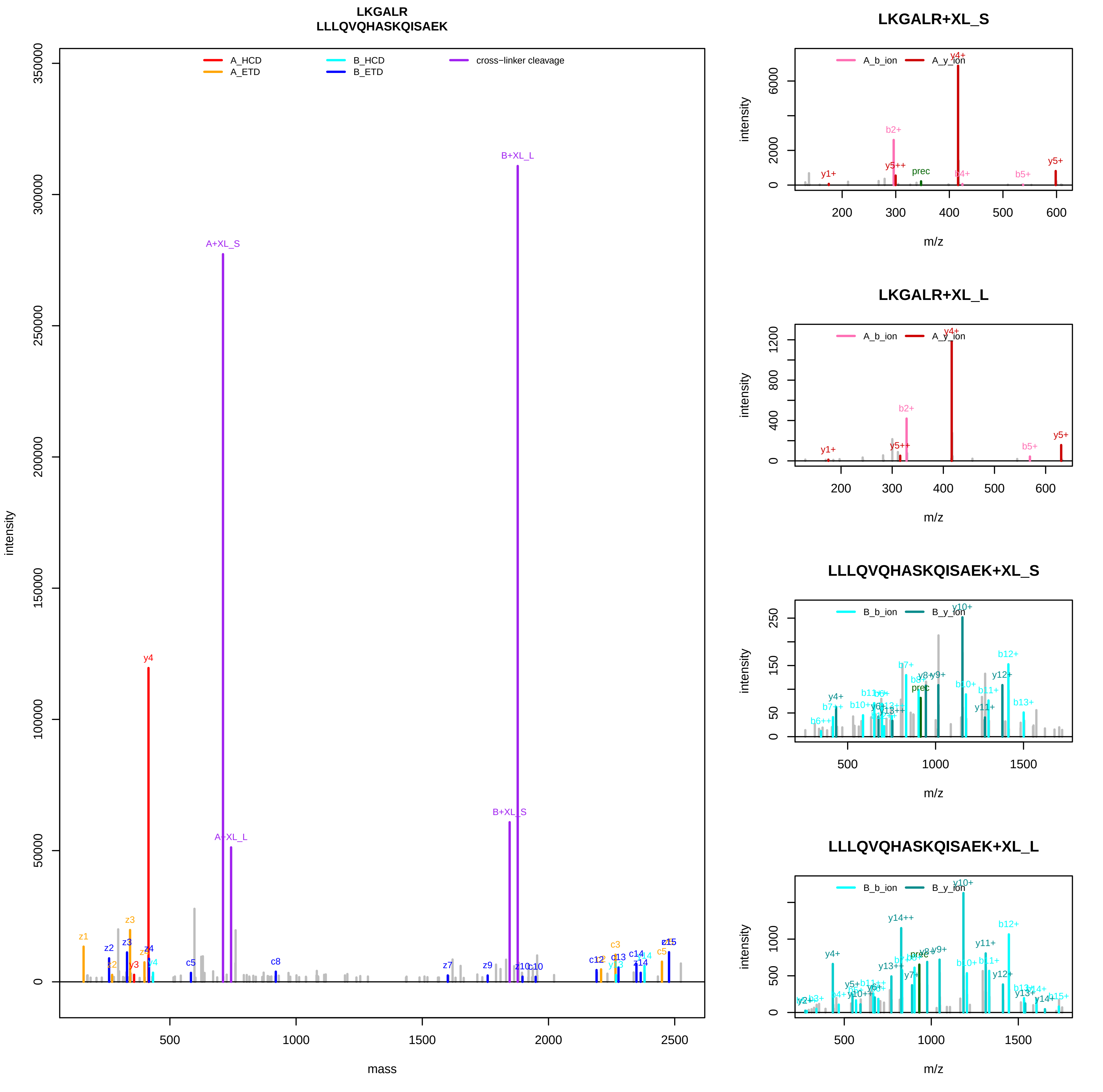

LKGALR
LLLQVQHASKQISAEK
A_HCD
A_ETD
B_HCD
B_ETD
cross−linker cleavage
intensity
mass
A+XL_S
A+XL_L
B+XL_L
B+XL_S
y4
y3
z1
z2
z3
z4
c5
c8
z7
z9
z10
c10
c12
c13
c14
z14
c15
c3
c5
LKGALR+XL_S
LKGALR+XL_L
LLLQVQHASKQISAEK+XL_S
LLLQVQHASKQISAEK+XL_L
A_b_ion
A_y_ion
B_b_ion
B_y_ion
m/z
prec
y1+
y4+
y5+
y5++
b2+
b4+
b5+
y10+
y12+
b12+
y11+
b10+
b11+
b7+
b13+
y14++
y13++
